# Maximising the potential of neuroimmunology

**DOI:** 10.1016/j.bbi.2020.03.010

**Published:** 2020-07

**Authors:** Lindsey J. Caldwell, Sumithra Subramaniam, Georgina MacKenzie, Divya K. Shah

**Affiliations:** Wellcome Trust, London, UK

## Abstract

Technological developments in recent years have led to a surge in advances in neuroimmunology, making real progress towards improving human health. With the scale of the challenges ahead, realising this potential requires a collaborative effort. The neuroscience, immunology and wider scientific community, both academia and industry, must come together to pool together ideas, experiences and resources.

## 1 Introduction

Neuroimmunology has great potential to impact on human health. Understanding immune involvement in regulating the nervous system, and vice versa, can provide effective strategies to diagnose and treat neurological and psychiatric conditions, from measuring immunological biomarkers for diagnosis, monitoring or patient stratification, to immune therapeutics. The field is now at a crossroads where the complexity and challenges of the scientific questions means that no single lab or discipline can tackle the problems alone.

## 2 Historical overview of neuroimmunology

Neuroimmunology emerged to study the intersection of the nervous and immune systems, combining the knowledge and techniques used by neuroscientists and immunologists. Clinical descriptions of neuroinflammatory disorders were documented as early as the 1600s, including Multiple Sclerosis (MS) (Landtblom, 2010) and Myasthenia Gravis (MG) (Conti-Fine et al., 2006), although involvement of the immune system was not appreciated until much later. As investigation into the pathological mechanisms underlying disease progressed, the role of the immune system became more evident. This was demonstrated by a number of discoveries in the mid-1900s, from the transfer of experimental autoimmune encephalitis (EAE; animal model of MS) by cells of the lymph node (Paterson, 1960), later identified as T-cells (Pettinelli and McFarlin, 1981), to autoantibodies against the acetylcholine receptor being linked to MG (Lindstrom, 1976).

However, early experiments on the blood–brain barrier (BBB) defined the immune privileged status of the brain, and this dogma limited momentum in the field for a long time. Over the past few decades, there has been mounting evidence to break down these early misconceptions. Technological improvements, from microscopy and tissue staining techniques, to high-resolution non-invasive imaging techniques (e.g. positron emission tomography; PET), have demonstrated the presence of immune cells and molecules in the brain (Nutma, 2019). In the 1980s, David Felten identified nerves innervating the lymph node and spleen, in direct contact with lymphocytes (Felten, 1985). Using a cyclophosphamide taste aversion learning paradigm, psychologist Richard Ader and immunologist Nicholas Cohen demonstrated that the immune system can be influenced by the brain, coining the term “psychoneuroimmunology” (Ader and Cohen, 1975). Taking this further, Ronald Glaser demonstrated the impact of behaviour, such as stress, on immune responses in various scenarios, from wound healing (Kiecolt-Glaser, 1995), to cancer progression (Andersen et al., 1994) and response to vaccinations (Glaser, 2000).

As progress in immunology moved forward our understanding of the different components and networks of the immune system, such as the identification of functional subsets of T-cells and microglia, their roles in neuroinflammatory diseases were also unravelled. This prompted new therapeutic approaches targeting the immune system, an early example of which includes plasma exchange to treat MG (Pinching and Peters, 1976). Recognition of the immune system’s involvement in a number of neurological disorders and mental health conditions has been growing ever since: from Alzheimer’s Disease (Van Eldik, 2016, Bryson and Lynch, 2016) and traumatic brain injury (McKee and Lukens, 2016) to schizophrenia (Khandaker, 2015) and depression (Leonard, 2010).

## 3 Future directions for neuroimmunology

Recent years have seen the field of neuroimmunology continue to expand rapidly (for recent reviews see (Nutma, 2019, Gruol, 2017, Bechter et al., 2019). Advances include discovery of a rudimentary meningeal lymphatic system and glymphatic system for waste clearance, which is changing our understanding of the crosstalk between the nervous and immune systems. Indeed, while interaction between the two systems in disease was recognised early on, we are now also starting to appreciate the role of the immune system in healthy brain development and homeostasis (Morimoto and Nakajima, 2019), and equally the importance of the nervous system in regulating the immune system (Ben-Shaanan, 2018).

The field is now at a tipping point, able to make the most of the recent technological developments, such as mass cytometry, single cell RNA sequencing, 2-photon microscopy, iPSCs and gene editing technologies. To capitalise on these developments and explore the current challenges and opportunities in the field, Wellcome held a meeting on neuroimmunology in June 2019, bringing together participants from academia, industry, funding bodies and charities. This was followed by a satellite meeting on psychosis, to focus more specifically on immunological biomarkers and potential interventions to support the ongoing work of the Wellcome Innovations Psychosis Flagship. The vision of the Flagship is to reduce the global burden of psychosis by supporting a portfolio of projects that will improve diagnosis, maximise the impact of early treatment and develop novel targeted interventions.

While the potential for neuroimmunology to influence how we approach neurological and psychiatric disorders was widely acknowledged, gaps remain in our basic understanding of the field. Addressing these issues and overcoming the disparity between the two disciplines requires a true collaborative effort, with members of the neuroscience and immunology communities, and academia and industry, coming together in partnership, in order to make real advances.

## 4 Neuroimmunology – an opportunity to impact on human health

With immune involvement increasingly recognised in neurological disorders and mental health conditions, there is an opportunity for neuroimmunology to impact on human health in a number of ways. Two key areas which are starting to show potential are: 1) immunological biomarkers and 2) therapeutics targeting the immune system.

Clinical diagnosis and management of neurological and psychiatric disorders remains a significant challenge. Due to the heterogenous nature of the population together with co-morbidities and challenges in directly accessing the central nervous system, diagnoses are often reliant on clinical interviews and subjective symptoms, particularly in psychiatry. While two patients may present with the same symptoms and be given the same diagnosis, they could have very different underlying pathophysiology. These barriers to prescribing the most effective treatment, as well as stratifying patients for clinical trials, has led to significant efforts towards biomarker discovery and validation. As our understanding of immune involvement in these disorders has grown, components of the immune system are being investigated as potential biomarkers to inform diagnosis, monitor diseases and treatment, or stratify patients. In psychosis for example, studies show a clear relationship between serious early-life infection and psychosis risk (Meyer and Feldon, 2009, Kappelmann, 2019, Khandaker, 2018, Khandaker, 2012). Increased levels of peripheral inflammation at psychosis onset (such as IL-6, IFN-γ, and cortisol) have been associated with clinical status and a lack of response to anti-psychotics (Mondelli, 2015). Activation of the peripheral immune system has also been associated with major depressive disorder (MDD), demonstrated by elevated levels of inflammatory markers, such as C-reactive protein (CRP). A greater difference was seen in treatment-resistant depression providing evidence that targeting the immune system may improve symptoms for this underserved patient group (Chamberlain, 2019). Given the potential impact to patients on diagnosis, prognosis and management, further research into immune biomarkers and how they relate to underlying physiology and brain function should be supported.

Targeting these inflammatory markers is an attractive therapeutic avenue that is gaining momentum. In MDD, increased CRP levels and treatment-resistance are also associated with other clinical aspects, including obesity, sleep disturbance and anxiety, suggestive of a clinical subgroup with an inflammatory phenotype (Chamberlain, 2019). Given that inflammatory markers, such as cytokines, were shown to impact on the mechanism of action of conventional anti-depressants (Zhu, 2010, Miller et al., 2009), a recent trial looked at the impact of anti-TNF-α therapy (Infliximab) on treatment-resistant depression (Raison, 2013). Whilst there was no difference in treatment outcomes between the placebo and treatment groups overall, symptomatic improvement was seen in patients with elevated pre-trial baseline CRP (Raison, 2013). Evidently there is more to understand about the role of inflammation in psychiatric disorders and treatment response. However, with over 20 ongoing trials currently looking at immune involvement in depression, this is a clear area of growth.

Depression and schizophrenia are still at relatively early stages of translating neuroimmunology to therapeutic impact, however MS has been leading the field in this area, with early therapies exhibiting a broad immune impact and resolving symptoms (e.g. corticosteroids), while more recent therapies have become increasingly targeted and specific e.g. monoclonal antibodies targeting B-cells (Ocrelizumab) (Rommer, 2019). Antigen-specific immunotherapy provides ultimate specificity, inducing protective immunity targeted to pathogenic T-cells, while avoiding non-specific immune suppression, but this requires careful selection of peptides (Anderton, 2002). Phase IIa studies of ATX-MS-1467 has shown promise in relapsing MS, with a 70% reduction in contrast-enhancing lesions at the end of the study and no evidence of unexpected safety signals (Chataway, 2018).

## 5 To improve the lives of patients we need to further our understanding of the basic mechanisms and function of brain – immune interactions

Realising the therapeutic potential outlined above requires a deeper understanding of how the nervous and immune systems interact. Here we have outlined three research themes in neuroimmunology, inspired by the meeting discussions, that should be prioritised to deliver health impact:

### 5.1 Where do the nervous system and immune system interact?

Mapping the anatomical and functional connections between the nervous and immune systems is essential to understanding how the two systems interact. The recent proposal of neuro-immune cell units goes someway to address this, describing discrete units representing defined anatomical locations where neurons and immune cells colocalise and functionally interact in tissues throughout the body, including lymphoid organs, adipose tissue, and mucosal barriers (Godinho-Silva et al., 2019). The extent to which the peripheral immune system directly impacts brain function requires a deeper understanding of how access of immune cells and molecules to the brain is regulated at the blood brain barrier (BBB). Which immune molecules and cells are able to cross the barrier? How does this change throughout the lifespan and across the course of various neurological and psychiatric conditions? How is the BBB affected in immune-mediated conditions such as in autoimmune disorders? Examining the indirect effects of the immune system on the brain, such as via visceral afferent fibres (Critchley and Harrison, 2013, Savitz and Harrison, 2018) and how these multiple parallel lines of communication intersect, will be crucial to gain a full picture of the interactions between the two systems.

### 5.2 What are the cells and molecules mediating neuro-immune interactions?

Identifying which immune cells in the periphery affect the brain is key to developing immune-based treatments for neurological conditions. Similarly, it is important to know which neurons affect immune cells, and to identify the cytokines and neurotransmitters mediating these interactions. Within the brain, we also need to understand the heterogeneity of microglia across brain regions and how this changes over time (Tan et al., 2019), reflects microglia function and compares to immune cells in the periphery. For example, microglia are differentially affected by ageing in a brain region-dependent manner (Grabert, 2016), suggesting immune-based therapeutic strategies could be used to target brain regions affected by neurodegenerative disorders. Meningeal lymphocytes have been implicated in social behaviour (Filiano, 2016), opening the doors for investigation of the role of immune cells in tissue surrounding the brain. Beyond neurons and microglia, it’s important to investigate how cells of the immune system interact with glia, including oligodendrocytes and astrocytes, and epithelial cells, as these cell types are critical for brain function (Barres, 2008). Understanding which cells and molecules mediate neuro-immune interactions in health is an essential baseline to investigate changes throughout the course of a disease/disorder.

### 5.3 How does the nervous system impact on the function of the immune system, and vice versa?

It is important to unpick when the immune system’s involvement in disorders such as Alzheimer’s disease and traumatic brain injury can be helpful and when it can be a hindrance. Understanding the dynamics and complexity of this question could uncover underlying disease mechanisms and lead to new diagnostic and prognostic tools as well as immune-based therapies for neurological conditions that have no effective treatment. Additionally, studying the role of the immune system in healthy brain development and homeostasis is important for understanding how this changes in neurodevelopmental disorders such as autism and schizophrenia (McAllister and Patterson, 2012). Likewise, investigating the functional effects of neurons on the immune system has the potential to deliver new therapeutic strategies. For example, harnessing the brain’s influence over the immune system could lead to non-pharmacological treatments, such as psychological therapies to impact the immune system to reduce tumours (Ben-Shaanan, 2018).

Unravelling these outstanding questions will require development of tools, techniques and methodologies (Box 1) in order to improve our understanding of the interaction of these two systems. As discussed earlier, the potential to harness the immune system to address neurological disorders has been acknowledged. Research into the influence of the immune system in brain health and disease has advanced in some areas, such that addressing these questions will help advance translation to impact on patient health. However, our knowledge of the influence of the nervous system on immune function is at an earlier stage of discovery and will require tackling some of the more fundamental questions outlined here. While this approach may have therapeutic potential in the future, we are just starting to scratch the surface of this up and coming area. Presumably findings from one aspect can be fed into the other and progress the field faster.

BOX 1 Developments needed to address the gaps in our fundamental understanding of neuro-immune interactions:

• Standardisation of iPSC-derived immune cell and neuron protocols
• *In vitro* systems to understand cell–cell interactions
• Access to patient samples (including blood, CSF, tissue)
• Standardisation of sample collection and storage
• Markers to identify different immune cell types and levels of activation in the brain
• Relevant animal models of disease
• Methods to manipulate the systemic immune system without affecting immune cells in the CNS and vice versa
• Non-invasive live imaging methods to visualise immune cells and the effects of inflammation at high resolution, in human and animal models in the CNS and PNS
• Experimental studies in humans to better understand how inflammation affects the brain, or vice versa
• Longitudinal cohort studies

## 6 Addressing these challenges requires a collaborative effort

These scientific challenges are broad and complex. Advancing our understanding of neuroimmune interactions requires close collaboration between neuroscientists and immunologists, in addition to expertise from other disciplines, e.g. imaging specialists, clinicians, allied health professionals, as well as bioinformaticians, engineers and researchers from the physical sciences. This can be particularly effective for disease-focused research, bringing together different expertise and disciplines to address the same problem. Cross-disease working however can also be beneficial where there are common basic mechanisms and pathways between multiple disorders, to identify these similarities and minimise duplication of effort.

Sharing knowledge, skills, and tools between academia and industry has the potential to accelerate the development of new treatments for disease. For example, in 2014 Wellcome funded the Neuroimmunology of Mood disorders and Alzheimer’s disease (NIMA) consortium, bringing together academic researchers and pharmaceutical companies to investigate the potential of targeting the immune system to treat these disorders in the brain, which has thus far resulted in the design of two ongoing clinical studies of immune-modulating therapies.

Researchers can find it challenging to branch into neuroimmunology because the disciplines of neuroscience and immunology have evolved independently, using different techniques and vocabulary. Bridging the gap to develop an understanding of the other discipline beyond nomenclature, cell types and their markers, requires closer working relationships and an immersion in the opposite field. This can be addressed with virtual institutes such as The Hodge Centre for Neuropsychiatric Immunology in Cardiff, or physical co-location of scientists from both disciplines, as seen in The Lydia Becker Institute of Immunology and Inflammation in Manchester.

The future of neuroimmunology will also be shaped by how we train and foster the next generation of scientists. Early exposure to neuroscience and immunology is key to attracting future researchers with an appreciation of both disciplines to the field of neuroimmunology. Our recently funded PhD programme in Neuroimmunology at King’s College London aims to train researchers in both disciplines, thereby strengthening neuroimmunology as its own field and growing the sense of community. Encouraging a mix of specialists and cross disciplinary trained researchers will ameliorate concerns that cultivating a monoculture of neuroimmunologists could isolate the field and prevent it from remaining at the cutting edge.

## 7 Conclusion

Neuroimmunology presents an opportunity to deliver new therapeutic approaches for a broad range of conditions, including many neurological and psychiatric conditions which have seen slow progress towards treatments in recent years. Following recent technological advances, realising the potential of neuroimmunology to impact human health now requires close collaboration between two disparate fields. The contribution of immunology in neuroscience is now well-accepted, whereas the impact of neuroscience on immunology is still in its infancy and an area of growth in coming years. This can be supported by formation of consortia, virtual and physical institutes, and encouraging training across disciplines. Partnerships with industry will be essential to make progress towards addressing the unknowns of neuro-immune interactions, including where, when, and how these two systems interact in health and disease. Answering these questions is key to enabling neuroimmunology research to progress fundamental mechanistic understanding, which in turn will pave the way to improving patient health.
